# Neuroimaging Correlates of Adolescent Substance Use: A Systematic Review of the Adolescent Brain Cognitive Development Study

**DOI:** 10.21203/rs.3.rs-11484087/v1

**Published:** 2026-10-27

**Authors:** Shervin Assari, Jordan Ray

**Affiliations:** Charles R. Drew University of Medicine and Science

**Keywords:** adolescent brain cognitive development, substance use, neuroimaging, magnetic resonance imaging, systematic review, adolescence

## Abstract

**Background:**

Neuroimaging may help characterize brain and contextual factors associated with substance use during development. The ABCD study supports investigation of developmental pathways relevant to substance use [1].

**Objective:**

To synthesize original ABCD publications examining MRI measures in relation to substance use and substance-related exposures.

**Methods:**

The authors screened all 2,700 publications in the ABCD corpus. Original empirical articles reporting an MRI measure of any modality and a substance use variable were eligible; methodological, conceptual, review, and commentary articles were excluded. Imaging modality, brain measures, substances, design, and association direction were extracted for descriptive synthesis.

**Results:**

The evidence considered spans morphology, white matter microstructure, resting-state connectivity, and task activation. Findings concerning youth initiation, prenatal exposure, and familial liability address distinct developmental questions. Family history and stressful life events feature in the white matter literature [2]. Findings vary by modality, exposure definition, and temporal direction, and include null associations.

**Conclusions:**

The literature suggests distributed and context-dependent neuroimaging associations. Observational designs, overlapping participants, confounding, and analytic heterogeneity limit causal interpretation and assessment of independent replication. The findings support further longitudinal investigation but do not establish a validated MRI screening tool.

## Introduction

Adolescent substance use is a public health concern because experimentation may be followed by continued use and related difficulties. Initiation, escalation, and substance use disorder are different outcomes and should not be treated as interchangeable. The developmental rationale for the ABCD study includes identifying brain and behavioral pathways relevant to prevention and treatment [1]. A systematic synthesis of its empirical neuroimaging literature can clarify which associations concern risk before initiation, which concern exposure, and where evidence remains limited.

### Brain development and substance use

Reward processing, cognitive control, and emotional regulation provide useful domains for studying substance use, although neuroimaging differences do not necessarily indicate impairment [3-5]. Nucleus accumbens volume has been examined in relation to tobacco or marijuana use following stressful life events [6]. White matter studies also offer relevant developmental context, but an association with executive functioning or psychopathology alone does not establish a substance use association or meet the eligibility criteria for this review [3].

The temporal ordering of imaging and substance measures is central to interpretation. Morphometry associated with polygenic liability may reflect characteristics present before youth exposure, whereas task activation associated with later initiation addresses prospective risk. Genetic-liability and emotional inhibitory-control research illustrate these distinct questions [4, 5]. Evidence concerning these domains requires interpretation within the cohort, exposure timing, and analytic design of each study.

### Family and environmental context

Neuroimaging associations occur within social and family contexts. Socioeconomic conditions, stress, and behavioral regulation may be related both to brain measures and to opportunities for substance use. Research examining pallidum connectivity and inhibitory control illustrates this approach [7]. Statistical mediation can describe an indirect association under model assumptions; it does not by itself demonstrate that disadvantage acts through a specific neural mechanism.

Prenatal exposure and youth substance use represent different exposure periods. Prenatal cannabis research examines maternal use during pregnancy in relation to offspring imaging measures [8]. Family history and stressful life events concern developmental context and liability [2]. Neither should be interpreted as evidence that a child has used substances. Separating these pathways prevents prenatal or familial findings from being misclassified as consequences of adolescent use.

### Rationale for the review

A central contribution of ABCD is the opportunity to examine developmental pathways relevant to substance use [1]. However, an association detectable before initiation is not automatically a specific risk marker, and a group difference is not necessarily useful for individual prediction. A systematic review should evaluate the timing, magnitude, consistency, and limitations of the evidence before drawing clinical conclusions.

Prospective functional-connectivity analyses can test whether baseline imaging is associated with later initiation [9]. Their value depends on the outcome definition, follow-up period, adjustment strategy, and validation design. These findings motivate synthesis across modalities, but they do not justify replacing established behavioral assessment with MRI or assuming that an imaging pattern is a modifiable treatment target.

### Review objectives

This review examines original empirical ABCD publications containing both MRI and substance use measures. Imaging methods described for ABCD provide methodological background [10]; methodological publications are not eligible evidence reports. The review asks which substances and exposure periods have been studied, which imaging measures have been examined, and how associations differ by study design and temporal direction.

The synthesis distinguishes youth substance use from prenatal exposure and familial substance-related liability. Within youth use, substance-specific outcomes should be separated from composite initiation measures, and experimentation should be distinguished from more sustained use. Cognitive and expectancy studies may inform interpretation [11], but they qualify for the imaging review only when an MRI measure is analyzed in relation to a substance variable.

The aim is to identify recurring findings, inconsistent or null results, and gaps that limit interpretation. Standardized imaging methods support comparability [12], but differences in analytic samples, preprocessing, outcomes, and statistical models still require explicit evaluation. Predictive modeling is considered a research implication rather than the organizing purpose of the review.

## Conceptual framework

### Exposure timing and substance-related liability

Substance-related liability can include family history, genetic propensity, behavioral traits, and environmental conditions. Family history and stress have been examined together in white matter research [2]. Studies of parental internalizing and externalizing problems [13] and broader parental psychopathology [14] provide developmental context but are not automatically substance-related. Parental problematic substance use has also been examined in relation to reward anticipation [15]. These categories should remain distinct because they involve different exposures and sources of confounding.

Stress may be associated with substance use and developmental imaging measures, although observational evidence requires cautious interpretation. Research on nucleus accumbens volume and stressful life events addresses variation in tobacco or marijuana use [6]; family-history research examines white matter measures [2]. These structural associations [6] and familial associations [2] do not establish that stress caused neural injury.

### Interpretation of MRI measures

Multimodal MRI can describe complementary aspects of brain development. The ABCD image-processing literature provides a basis for understanding these measures [12]. Structural morphology, diffusion-derived metrics, resting-state connectivity, and task activation are not interchangeable indicators of brain health. Each requires interpretation in relation to acquisition, preprocessing, reliability, and the developmental stage of participants.

Structural MRI quantifies features such as volume, cortical thickness, and surface area. Translating imaging measures into clinical applications requires attention to validation and responsible deployment **[**16**]**. Nucleus accumbens volume has been examined in relation to stress and later tobacco or marijuana use [6]. Parental psychopathology studies offer structural context [13]. Regional differences should be described as differences rather than deficits unless functional impairment is demonstrated.

Functional MRI measures blood-oxygen-level-dependent signals. Resting-state analyses summarize statistical coupling across regions; task analyses examine activity or connectivity during defined conditions. Genetic-liability research should not be used as a substitute for direct functional evidence [5]. Emotional inhibitory-control research [4] and pallidum connectivity research [7] illustrate different analytic questions, with different requirements for cohort verification, task identification, and temporal interpretation.

Diffusion MRI characterizes aspects of water diffusion and is sensitive to white matter microstructure. Associations involving executive functioning and psychopathology provide relevant context [3], while family-history and stress studies address substance-related liability [2]. Fractional anisotropy and diffusivity are not specific measures of tissue damage. Their meaning depends on anatomy, development, acquisition, and model assumptions.

### Distinguishing association from prediction

Repeated assessments help establish measurement order, although observational follow-up does not remove confounding. Neurocognitive trajectory research using self-report and toxicology illustrates the importance of exposure ascertainment [17]. Pre-initiation imaging differences require documentation that youth had not initiated use when imaging was obtained.

Methodological articles remain useful for interpreting data acquisition and processing [12], but they are excluded from the empirical synthesis. A report must analyze ABCD data, include an MRI measure, and include a substance variable relevant to the review question. The presence of a brain-related concept, a cognitive task, or a general risk factor is insufficient.

Standardized image-processing methods provide the methodological basis for interpreting ABCD MRI measures [12].

## Methods

### Review design and information source

We conducted a systematic review of original empirical publications using ABCD data. The authors screened all 2,700 publications in the ABCD corpus available at the review cutoff, rather than restricting the initial corpus by imaging or substance keywords. Methodological, conceptual, review, and commentary papers were excluded.

### Eligibility criteria

Eligible reports were original empirical articles that analyzed ABCD participants and included at least one MRI-derived measure of any modality and at least one substance use variable. Eligible imaging measures could include structural morphology, diffusion-derived measures, resting-state connectivity, or task-related activation or connectivity. Cognitive or behavioral measurements without MRI analysis did not satisfy the imaging criterion. Studies using other cohorts did not satisfy the ABCD criterion.

The review covered any substance rather than only alcohol, cannabis, and tobacco. Youth substance use, prenatal exposure, and familial or genetic liability were distinguished in the narrative because they address different developmental questions. General parental psychopathology or socioeconomic disadvantage alone did not satisfy the substance criterion.

We excluded methodological and conceptual publications, reviews, and commentaries even when they discussed ABCD imaging or substance use.

### Screening and selection

All 2,700 publications were screened for eligibility. Multiple articles from ABCD represent separate reports, but may include overlapping participants and therefore do not constitute independent participant samples.

### Data extraction

Information on imaging modality, brain measures, substance variables, study design, and association direction was extracted. These characteristics were used to organize the descriptive synthesis and distinguish concurrent associations from prospective initiation and exposure-related imaging change.

### Risk of bias and confidence in the evidence

Interpretation considered potential selection into analytic samples, missing data and attrition, exposure misclassification, imaging motion and quality, confounding, multiple comparisons, selective reporting, and validation leakage. No formal study-level risk-of-bias ratings are presented, which limits assessment of confidence in the evidence.

### Evidence synthesis

Findings were summarized descriptively by exposure domain, modality, and temporal direction. Concurrent imaging–use associations, baseline imaging associated with later initiation, and use associated with later imaging change address different questions. Null results were distinguished from inverse associations.

No pooled estimate is presented. Differences in measures and outcomes, together with overlapping ABCD samples, limit quantitative aggregation. Article sample sizes cannot be summed to estimate the number of unique children represented in the literature.

## Results

### selection and characteristics

The authors screened 2,700 ABCD publications. Table 1 presents 26 reports considered in the evidence inventory, including contextual reports. Clinical artificial-intelligence guidance provides background on translation [18]; morphometry associated with polygenic substance-related liability addresses a distinct empirical question [5]. Contextual reports are distinguished from direct evidence connecting MRI with substance variables in ABCD.

Related background literature [1-9, 11-15, 17, 19, 20, 22-36].

Research involving adolescents with substance use disorder and unaffected siblings provides broader context on familial neuroanatomical patterns [19]. Such findings should not be generalized to ABCD participants without evidence that they arise from the same cohort and comparable exposure definitions.

### Structural and diffusion MRI

Structural and diffusion research addresses regional morphology and white matter measures. Executive-function and psychopathology findings provide developmental context [3], while substance-related analyses address more specific exposures. Heterogeneity in metrics, contrasts, and adjustment limits conclusions about a consistent structural pattern or overall effect magnitude.

Diffusion-derived metrics require interpretation in relation to the tract, developmental stage, and exposure examined. White matter associations with executive functioning or psychopathology [3] do not by themselves establish a substance-specific risk threshold. Prospective youth-use associations, familial liability, and prenatal-exposure comparisons address different questions.

### Resting-state functional connectivity

Resting-state evidence includes analyses asking whether connectivity measured at baseline is associated with subsequent initiation [9]. Such studies concern temporal prediction of behavior, rather than exposure-related change in connectivity. Reported predictive associations should be interpreted alongside the validation approach and the contribution of demographic, behavioral, and environmental variables.

Morphometry associated with polygenic substance-related liability [5] concerns structural variation and cannot establish functional-network differences. Structural and functional measures provide complementary information but should be interpreted according to the modality actually analyzed.

Hippocampal functional connectivity has been examined in relation to impulsivity and subsequent substance use [20]. Concurrent imaging–behavior associations and later substance outcomes may occur within the same report. Measurement timing is therefore more informative than a single cross-sectional or longitudinal label.

Whole-brain connectivity research has examined shared signatures involving impulsivity, neuroticism, and alcohol-use risk [21]. These associations raise questions about specificity: a connectivity pattern may relate to several behavioral traits rather than to substance use alone. Independent estimates of the imaging–use association and the imaging–trait association are needed before inferring a shared mechanism.

Whole-brain connectivity and behavioral-trait findings raise questions about specificity and prediction [22]. High-dimensional imaging data alone do not establish improved individual prediction. Models require evidence of performance beyond simpler measures, calibration, reproducibility, and generalizability.

### Prenatal exposure and contextual associations

The prenatal cannabis literature includes structural and diffusion measures in late childhood [8]. These reports address associations between prenatal exposure and offspring imaging, rather than the consequences of youth cannabis use. Interpretation must account for exposure timing, ascertainment, co-exposures, and family or social characteristics that may differ between exposed and unexposed children.

Hippocampal connectivity findings concern memory-related circuitry, impulsivity, and substance use [20]. These associations should remain distinct from pallidum mediation analyses. Indirect associations do not establish a causal neural pathway linking socioeconomic conditions with initiation.

Family history of substance use and stressful life events have been investigated in relation to cerebral white matter [2]. These results concern contextual and familial liability. Unless repeated within-person imaging was analyzed, differences across age cannot be treated as observed developmental change. The extracted design and measurement schedule should determine the language used to describe maturation.

Sensorimotor connectivity research connects imaging with several social and behavioral measures [23]. Such breadth may indicate shared correlates rather than a substance-specific signature. These findings should be synthesized separately from prenatal exposure and direct youth-use analyses, with attention to covariate adjustment, measurement timing, and the distinction between mediation and association.

A null association is an informative result, not a negative-direction association. Patel and colleagues examined whether potentially traumatic events and early substance use predicted changes in resting-state connectivity [24]. The reported absence of detectable prediction [24] does not directly contradict Kardan and colleagues, who examined baseline connectivity in relation to later initiation [9]. The studies address different temporal directions and outcomes; comparison should consider exposure prevalence, follow-up, effect estimates, and statistical precision.

## Discussion

### Principal findings and interpretation

The literature addresses substance-related questions across several imaging modalities and exposure periods. It does not establish a single recurring neural pattern or an overall effect magnitude. Neurocognitive trajectory research provides broader developmental context [17]. Separating pre-initiation correlates, exposure-related imaging differences, and prenatal or familial associations is central to interpretation.

Exposure measurement is a major source of uncertainty. Self-report and toxicology capture different aspects and time windows of use [17]. Misclassification may obscure timing or weaken associations. Definitions of a sip or puff, repeated use, composite initiation, and substance-specific exposure can substantially affect comparability.

### Confounding and specificity

Family history, stressful life events, and brain measures may share determinants [2]. Adjustment cannot guarantee removal of confounding, especially when social context, parental mental health, co-exposure, and behavioral traits are measured incompletely. A pre-initiation association may therefore reflect common liability or contextual conditions rather than a direct neural contribution to use. Genetic-liability analyses also require careful interpretation of population structure and transferability.

Parental psychopathology is relevant because it may be associated with offspring brain structure and with family circumstances [14]. A substance-related imaging association may overlap with broader internalizing or externalizing vulnerability. Specificity should be assessed through clearly defined exposures and comparison outcomes, rather than inferred from the presence of a significant association.

### Limitations of this review

This review is bounded by a single cohort and its publication inventory. Findings cannot automatically be generalized beyond the populations, age range, exposure levels, and imaging protocols represented in ABCD. Published findings may be affected by selective reporting. Shared participants, heterogeneous endpoints, and limited comparable effect estimates constrain assessment of independent replication and overall effect size.

The descriptive synthesis does not provide pooled effects or formal study-level risk-of-bias ratings. Confidence in the evidence therefore remains limited, particularly where measurement timing, exposure definitions, and analytic samples differ across reports.

### Research priorities

#### Longitudinal and reproducible analyses

The developmental goals of ABCD support research that follows youth across initiation and changing patterns of use [1]. Future work should distinguish any initiation from sustained exposure and investigate within-person imaging trajectories where repeated scans are available. Consistent exposure definitions, transparent preprocessing, and sensitivity analyses would improve comparison across reports.

Adams and colleagues examined inhibitory-control activation in relation to early low-level alcohol initiation in ABCD [25]. Reported differences during correct-stop contrasts involved the right paracentral lobule and left isthmus gyrus. These study-specific findings do not establish intervention targets or clinically useful thresholds.

The broad range of associations examined in sensorimotor-connectivity research [23] supports investigating social context alongside imaging. Analyses should test whether imaging adds information beyond stress, socioeconomic conditions, impulsivity, and other readily assessed factors. Addressing adverse circumstances remains relevant regardless of whether a neural indirect association is observed.

#### Prediction and validation

ABCD processing methods support analysis of multiple imaging modalities [12], but combining modalities does not necessarily improve prediction. Candidate models should be compared with transparent baseline models and evaluated using data not used for feature selection or tuning. Family membership, site, repeated scans, and overlapping participants must be handled so that information does not leak across training and evaluation samples.

Nucleus accumbens research illustrates the importance of context and possible effect modification [6]. Before a measure is described as a marker of individual risk, research should demonstrate reproducibility, calibration, clinically meaningful discrimination, and transportability. Prespecified subgroup analyses may clarify heterogeneity, but subgroup differences should be supported by interaction tests and adequate precision.

Prospective connectivity findings provide a basis for further predictive research [9]. Neither statistical significance nor a high-dimensional representation is sufficient for clinical implementation. The added value of MRI should be demonstrated against less burdensome assessments, and any model should undergo external validation and assessment of potential harms before being proposed for routine screening.

### Clinical and public health implications

#### Implications for prevention

Familial neuroanatomical patterns offer contextual evidence on substance-related vulnerability [19]. The current synthesis does not establish an MRI-based screening indication. Imaging differences should not be used to label children as destined for substance use or attribute behavior to a fixed biological vulnerability.

Prenatal cannabis imaging research addresses a distinct developmental exposure [8]. It should not be extrapolated directly to the effects of adolescent experimentation. Counseling and prevention implications should reflect the exposure actually studied, the observational design, and the uncertainty of the association. Brain differences alone do not establish future impairment or a specific response to intervention.

#### Equity and ethical considerations

The ABCD ethics literature addresses oversight in multisite neuroimaging research involving children and adolescents [26]. Future predictive applications would require additional consideration of privacy, assent and consent, interpretability, and consequences of risk classification. Population associations should not be used to make deterministic judgments about an individual child.

Protocols for incidental findings remain relevant to pediatric MRI research [26]. Any proposed risk model should also be evaluated for unequal performance and unequal access across social groups and sites. Social adversity should be understood as a condition that may warrant support, not as an inherent neural deficit. Prevention implications should include supportive family, school, and community environments without making services contingent on MRI findings.

## Conclusion

The ABCD literature provides opportunities to examine substance-related brain associations alongside family history and stressful life events [2]. These factors may be interconnected, but the available observational evidence does not identify a single causal pathway. A credible synthesis requires explicit separation of youth use, prenatal exposure, and familial or genetic liability.

Morphometry associated with polygenic liability illustrates how substance-related questions can be investigated before youth exposure [5]. Structural, functional, and diffusion findings require interpretation in relation to study design and measurement timing. Their heterogeneity limits conclusions about a single neural signature.

The developmental rationale for ABCD remains relevant to prevention research [1]. This literature can guide better designed longitudinal studies. Current findings should be treated as research associations rather than validated screening markers or proof of substance-related brain injury.

## Figures and Tables

**Table:** 

First Author	Substance domain	MRI or other measure	Reported design	Reported finding	Linked citation
Adams 2026	Alcohol	Task-based fMRI [25]	Longitudinal	Youth who go on to endorse alcohol experimentation display significantly higher activation in the right paracentral lobule and the left isthmus gyrus during correct stop contrasts compared to alcohol naive youth.	[25]
Kardan 2024	General substance initiation	Resting-state fMRI	Longitudinal	Functional connectivity predicted prospective substance use initiation and was correlated with environmental factors.	[9]
Hiraoka 2023	Prenatal cannabis	Structural MRI (Brain volumes)	Longitudinal	Prenatal cannabis exposure is associated with differences in the developmental trajectories of cognitive ability and brain volumes.	[27]
Faraj 2022	Prenatal cannabis	Resting-state fMRI	Cross-sectional	Prenatal cannabis exposure is associated with resting-state functional connectivity alterations in the salience network.	[28]
Acosta 2025	Prenatal cannabis	Structural/Diffusion MRI	Longitudinal	Prenatal marijuana exposure is associated with differences in children's white matter microstructure and cortical morphology during late childhood.	[8]
Jones 2026	Cannabis, alcohol	Behavioral / Cognitive	Longitudinal	Youth cannabis and alcohol expectancies mediate associations between pre-adolescent cognitive function and subsequent use initiation.	[11]
Albar 2022	Indirect (risk context)	Structural MRI	Cross-sectional	Parental internalizing and externalizing behavior problems are associated with differences on children's limbic brain structures.	[13]
Wade 2026	Cannabis	Cognitive/Toxicology	Longitudinal	Assessed the influence of adolescent cannabis use onset on longitudinal neurocognitive trajectories using self-report combined with toxicology.	[17]
Ma 2025	SUD (Family History)	Diffusion MRI (White matter)	Cross-sectional	Family history of substance use disorders and stressful life events are associated with differences in the adolescent maturation of cerebral white matter.	[2]
Cheng 2026	Alcohol	Resting-state fMRI	Longitudinal	Impulsivity and neuroticism share distinct whole-brain functional connectivity signatures associated with youth alcohol-use risk.	[22]
Patel 2026	General substance use	Resting-state fMRI	Longitudinal	Potentially traumatic events and early substance use were not found to predict changes in resting-state functional connectivity in early adolescence.	[24]
Kayis 2025	General SUD	Structural MRI	Cross-sectional	Reports lower cortical thickness in the right inferior frontal cortex in adolescents with SUD and their unaffected biological siblings compared to controls.	[19]
Sneider 2025	General substance use	Task-based fMRI	Longitudinal	Adolescents who later initiate substance use show distinct baseline network signatures in systems supporting spatial memory, attention, and salience detection prior to initiation.	[29]
Flannery 2025	General substance use	Structural MRI (Tissue Iron)	Longitudinal	Structural changes in dopaminergic functioning are associated with adolescent substance use initiation and cognitive control.	[30]
Kramer 2025	Cannabis, General SU	Structural MRI	Cross-sectional	Elevated polygenic risk scores for substance use are linked to atypical morphometry (structural deviations) in the nucleus accumbens and hippocampus.	[5]
Sutherland 2025	General substance use	Task-based fMRI working memory task	Longitudinal	Working memory-related brain activations and deactivations are linked to adolescent substance use via alexithymia.	[31]
Wei 2026	General substance use	Structural MRI	Longitudinal	Frontal structural and microstructural phenotypes partially mediate the robust association between externalizing polygenic liability and substance use initiation. Publication status: preprint.	[32]
Cohen 2025	General substance use	Task-based fMRI (Go-NoGo)	Longitudinal	Baseline large-scale brain network recruitment during emotional inhibitory control tasks correlates with the risk of adolescent substance use initiation, revealing divergent pathways for suppressing impulses.	[4]
Navarro 2025	General substance use	Task-based fMRI (Reward)	Longitudinal	Parental history of problematic substance use is associated with pre-exposure differences in reward-processing brain regions, such as the striatum.	[15]
Assari 2025	General substance use	Resting-state fMRI	Cross-sectional	Baseline brain-wide resting-state functional connectivity of the hippocampus is associated with impulsivity and subsequent substance use.	[20]
Assari 2024	Tobacco, Marijuana	Structural MRI (NAc volume)	Longitudinal	Variations in nucleus accumbens volume are associated with youth tobacco and marijuana experimentation following exposure to stressful life events.	[6]
Assari 2021	Indirect (risk context)	Task-based fMRI (N-Back)	Cross-sectional	Parental education correlates with left lateral orbitofrontal cortical activity during cognitive working memory tasks.	[33]
Assari 2021	Indirect (risk context)	Structural MRI	Cross-sectional	Investigated sex differences in the association between hippocampal volume and working memory capacity in children.	[34]
Assari 2025	General substance use	Resting-state fMRI	Cross-sectional	Baseline resting-state sensory-motor connectivity is associated with socioeconomic disadvantage to psychosocial stress, impulsivity, and subsequent substance use in children.	[23]
Assari 2025	Tobacco, Marijuana	Resting-state fMRI	Cross-sectional	Pallidum functional hypoconnectivity (reduced connectivity) partially mediates the relationship between environmental influences (like low SES) and tobacco/marijuana initiation.	[7]
Mattoni 2023	Indirect (risk context)	Structural MRI	Cross-sectional	Multiple forms of parental psychopathology are associated with widespread pre-exposure alterations in offspring subcortical brain volumes.	[14]
